# Large-scale genetic investigation reveals genetic liability to multiple complex traits influencing a higher risk of ADHD

**DOI:** 10.1038/s41598-021-01517-7

**Published:** 2021-11-19

**Authors:** Luis M. García-Marín, Adrián I. Campos, Gabriel Cuéllar-Partida, Sarah E. Medland, Scott H. Kollins, Miguel E. Rentería

**Affiliations:** 1 Department of Genetics and Computational Biology, QIMR Berghofer Medical Research Institute, Brisbane, QLD Australia; 2 School of Biomedical Sciences, Faculty of Medicine, The University of Queensland, Brisbane, QLD Australia; 3 The University of Queensland Diamantina Institute, The University of Queensland, Woolloongabba, QLD Australia; 4 Department of Psychiatry & Behavioral Sciences, School of Medicine, Duke University, Durham, NC USA; 5 Holmusk Technologies, Inc., New York, NY USA; 6 Present Address: 23andMe, Inc, Sunnyvale, CA USA

**Keywords:** Genetics, Behavioural genetics, Genetic association study, Genetic linkage study, Neurodevelopmental disorders

## Abstract

Attention Deficit-Hyperactivity Disorder (ADHD) is a complex psychiatric and neurodevelopmental disorder that develops during childhood and spans into adulthood. ADHD’s aetiology is complex, and evidence about its cause and risk factors is limited. We leveraged genetic data from genome-wide association studies (GWAS) and performed latent causal variable analyses using a hypothesis-free approach to infer causal associations between 1387 complex traits and ADHD. We identified 37 inferred potential causal associations with ADHD risk. Our results reveal that genetic variants associated with *iron deficiency anemia (ICD10)*, *obesity*, *type 2 diabetes, synovitis and tenosynovitis (ICD10)*, *polyarthritis (ICD10)*,* neck or shoulder pain*, and *substance use* in adults display partial genetic causality on ADHD risk in children. Genetic variants associated with ADHD have a partial genetic causality increasing the risk for *chronic obstructive pulmonary disease* and *carpal tunnel syndrome.* Protective factors for ADHD risk included genetic variants associated with the likelihood of participating in socially supportive and interactive activities. Our results show that genetic liability to multiple complex traits influences a higher risk for ADHD, highlighting the potential role of cardiometabolic phenotypes and physical pain in ADHD’s aetiology. These findings have the potential to inform future clinical studies and development of interventions.

## Introduction

Attention Deficit-Hyperactivity Disorder (ADHD) is a complex neurodevelopmental and psychiatric disorder characterised by impulsivity, hyperactivity, and attention and concentration impairment^1,2^. It typically develops during childhood with symptoms often persisting to adulthood^3^, affecting around 5% of children and 2.5% of adults^4^.

ADHD is heritable, and its aetiology involves a variety of genetic and environmental factors. Twin and family studies have estimated ADHD heritability between 0.72 and 0.88^5^, positioning it among the most heritable psychiatric conditions. Genome-wide association studies (GWAS) have identified 304 genetic variants across 12 independent loci associated with ADHD^6^. Similarly, genetic correlations have been identified between ADHD and smoking, obesity-related phenotypes, and psychiatric disorders such as major depressive disorder and schizophrenia^6^.

The causal architecture of a polygenic phenotype pinpoints potential exposures and outcomes in putative causal pathways underlying the aetiology of a complex trait^7^. The heterogeneity and multifactorial causal architecture of ADHD, combined with the relatively small sample sizes of ADHD studies, pose challenges to identify potentially modifiable risk factors for ADHD^4^. Previous studies have identified shared genetic pathways between ADHD and other phenotypes^6^; however, a genetic correlation between two polygenic phenotypes is not sufficient evidence for a causal association. For instance, a genetic correlation could be explained by horizontal pleiotropic effects (i.e., genetic variants have a direct effect on both traits; therefore, a directional causal effect between the two traits does not exist), which in turn may produce spurious findings in genetic epidemiological studies investigating causality with traditional methods such as Mendelian randomisation^8^. Likewise, sample overlap between GWAS can bias results increasing false-positive findings in Mendelian randomisation studies^9^.

In the present study, we use the latent causal variable (LCV)^8^ method, which is less susceptible to bias by horizontal pleiotropy and sample overlap than Mendelian randomisation^8^, to conduct a large-scale genetic investigation of the causal architecture of ADHD. To this end, we conducted a hypothesis-free phenome-wide screening of traits causally associated with ADHD using the ADHD GWAS summary statistics from the Psychiatric Genomics Consortium (PGC) and an extensive collection of GWAS summary statistics for 1,387 other traits. Our results should be interpreted as a set of testable hypotheses for future epidemiological and interventional studies.

## Results

We identified 578 significant genetic correlations between ADHD risk and other complex traits (FDR < 5%; Supplementary File 1). Of those, 37 showed robust evidence of partial genetic causality with ADHD (|GCP|> 0.60; FDR < 5%) and an additional nine showed evidence for limited partial genetic causality (|GCP|< 0.60; FDR < 5%; Supplementary File 1).

**Figure 1:**
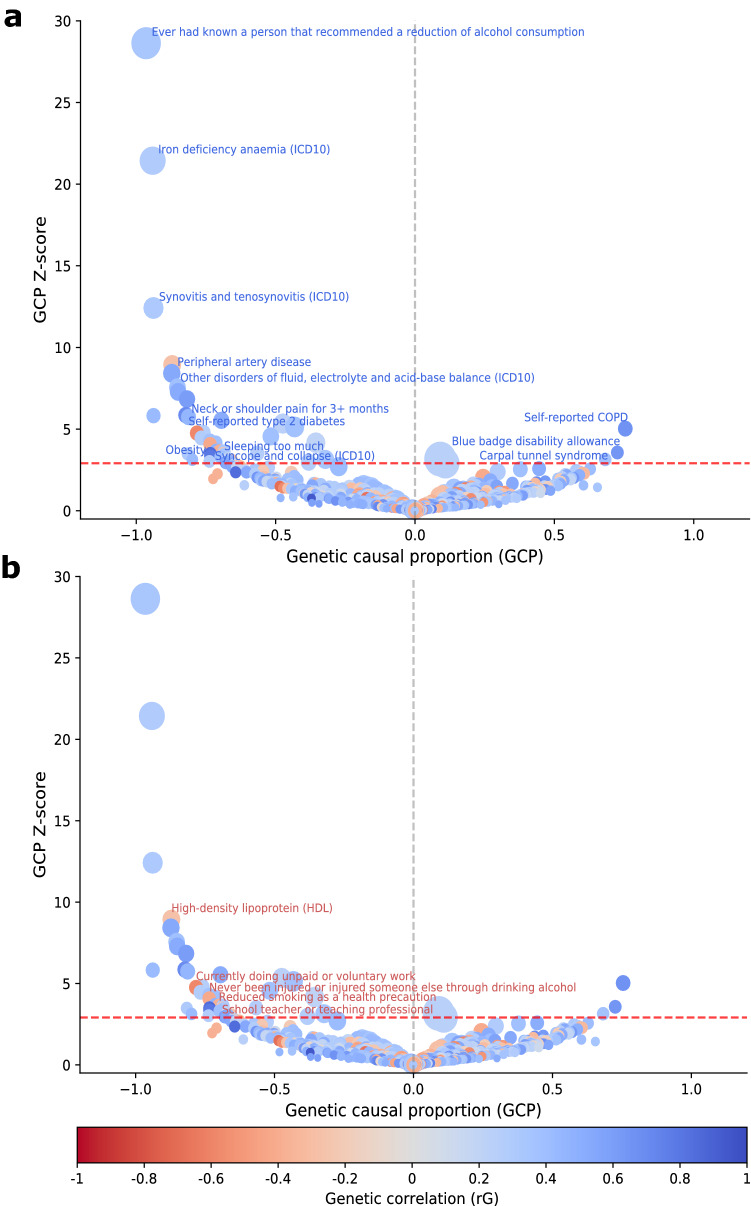
Causal architecture of ADHD. Causal architecture plots illustrating results from the phenome-wide analysis. Each dot represents a trait with genetic overlap with ADHD. The x-axis shows the GCP estimate, and the y-axis shows the genetic causal proportion (GCP) absolute Z-score (as a measure of statistical significance). The red dashed lines represent the statistical significance threshold (FDR < 5%). The division for traits causally influencing ADHD (on the left) and traits causally influenced by ADHD (on the right) is represented by the grey dashed lines. Results are shown separately for traits with a positive genetic correlation with ADHD (**a**) and a negative genetic correlation with ADHD (**b**). A GCP = 0 indicates that horizontal pleiotropic effects mediate the genetic correlation (i.e., provides no evidence for genetic causality between the phenotypes), whereas a |GCP| = 1 represents full genetic causality. A |GCP| < 0.60 represents limited partial genetic causality. A detailed description of how to interpret these plots is available in previous studies^46,47^.

Genetic variants associated with ADHD risk displayed positive genetic correlations and significant causal proportion on three complex traits (Fig. 1 and Table 1). Specifically, our results suggest that genetic liability for ADHD also increases risk of *self-reported chronic obstructive pulmonary disease (COPD)* (r_G_ = 0.67, GCP = 0.75, GCP_pvalue_ = 4.90 × 10^−07^), having a *blue badge disability allowance* (r_G_ = 0.64, GCP = 0.73, GCP_pvalue_ = 3.58 × 10^−04^) and presenting *carpal tunnel syndrome* (r_G_ = 0.37, GCP = 0.68, GCP_pvalue_ = 1.66 × 10^−03^) as an adult.

**Table 1:** Traits influenced by genetic liability to ADHD.

Trait	GCP	GCP se	GCP pval	r_G_	r_G_ se	r_G_ pval
Self-reported COPD	0.75	0.15	4.90E−07	0.67	0.14	2.71E−06
Blue badge disability allowance	0.73	0.20	3.58E−04	0.64	0.06	1.38E−29
Carpal tunnel syndrome	0.68	0.22	1.66E−03	0.37	0.06	2.63E−09

This table shows significant (FDR < 5%) traits with a robust and positive genetic causal proportion (GCP > 0.60) and a positive genetic correlation with ADHD. *Trait* Trait causally associated with ADHD, *GCP* Genetic causal proportion, *GCP se* Genetic causal proportion standard deviation, Genetic causal proportion, *GCP pval* Genetic causal proportion p-value before FDR correction, *r*_*G*_ Genetic correlation, *r*_*G*_* se* Genetic correlation standard deviation, *r*_*G*_* p val* Genetic correlation p-value before FDR correction. P-values after FDR correction are available for all traits in Supplementary File 1.

Genetic liability for *high-density lipoprotein (HDL)* (r_G_ = − 0.27, GCP = − 0.87, GCP_pvalue_ = 3.86 × 10^−19^), being a *school teacher or teaching professional* (r_G_ = − 0.26, GCP = − 0.69, GCP_pvalue_ = 5.28 × 10^−04^) and *doing unpaid or voluntary work* (r_G_ = − 0.59, GCP = − 0.78, GCP_pvalue_ = 1.96 × 10^−06^) as an adult displayed negative genetic correlations and significant genetic causal proportion with a high ADHD risk as a child (Table 2).

**Table 2:** Traits with a negative genetic correlation and significant partial causality on ADHD risk.

Trait	GCP	GCP se	GCP pval	r_G_	r_G_ se	r_G_ pval
High-density lipoprotein (HDL)	− 0.87	0.10	3.86E−19	− 0.27	0.06	1.23E−06
Doing unpaid or voluntary work	− 0.78	0.16	1.96E−06	− 0.59	0.10	1.66E−08
Never been injured or injured someone else through drinking alcohol	− 0.74	0.18	4.70E−05	− 0.42	0.18	1.76E−02
Reduced smoking as a health precaution	− 0.70	0.19	2.77E−04	− 0.38	0.15	1.28E−02
School teacher or teaching professional	− 0.69	0.20	5.28E−04	− 0.26	0.11	1.49E−02
Primary and nursery education teaching professionals	− 0.66	0.21	2.17E−03	− 0.26	0.11	1.63E−02

This table shows significant (FDR < 5%) traits with a robust genetic causal proportion (|GCP| > 0.60) and a negative genetic correlation with ADHD. *Trait* Trait causally associated with ADHD, *GCP* Genetic causal proportion, *GCP se* Genetic causal proportion standard deviation, Genetic causal proportion, *GCP pval* Genetic causal proportion p-value before FDR correction, *r*_*G*_ Genetic correlation, *r*_*G*_* se* Genetic correlation standard deviation, *r*_*G*_* p val* Genetic correlation p-value before FDR correction. P-values after FDR correction are available for all traits in Supplementary File 1.

We observed that genetic liability for a number of physical health conditions, psychiatric-related traits, and lifestyle-related phenotypes may increase ADHD risk as a child (Table 3 and Fig. 1). In particular, physical health conditions included cardiometabolic diseases such as iron deficiency *anaemia (ICD10)* (r_G_ = 0.29, GCP = − 0.94, GCP_pvalue_ = 6.25 × 10^−102^), *peripheral artery disease* (r_G_ = 0.54, GCP = − 0.87, GCP_pvalue_ = 3.76 × 10^−17^), *self-reported type 2 diabetes* (r_G_ = 0.45, GCP = − 0.81, GCP_pvalue_ = 9.44 × 10^−09^) and *obesity* (r_G_ = 0.86, GCP = − 0.73, GCP_pvalue_ = 4.88 × 10^−04^). Musculoskeletal-related traits, included *synovitis and tenosynovitis (ICD10)* (r_G_ = 0.32, GCP = − 0.94, GCP_pvalue_ = 2.11 × 10^−35^), *neck or shoulder pain for more than three months* (r_G_ = 0.70, GCP = − 0.82, GCP_pvalue_ = 4.63 × 10^−09^), *polyarthrosis (ICD10)* (r_G_ = 0.52, GCP = − 0.69, GCP_pvalue_ = 3.07 × 10^−08^) and *fibroblastic disorders* (r_G_ = 0.19, GCP = − 0.67, GCP_pvalue_ = 1.50 × 10^−03^). Finally, we observed a similar effect for genetic components associated with eye diseases such as *entropion and trichiasis of the eyelid* (r_G_ = 0.43, GCP = − 0.94, GCP_pvalue_ = 5.72 × 10^−09^).

**Table 3:** Traits with a positive genetic correlation and significant partial causality on ADHD risk.

Trait	GCP	GCP se	GCP pval	r_G_	r_G_ se	r_G_ pval
Ever had known a person that recommended a reduction of alcohol consumption	− 0.96	0.03	2.87E−180	0.34	0.12	5.94E−03
Iron deficiency anaemia (ICD10)	− 0.94	0.04	6.25E−102	0.29	0.10	3.23E−03
Synovitis and tenosynovitis (ICD10)	− 0.94	0.08	2.11E−35	0.32	0.12	6.15E−03
Peripheral artery disease	− 0.87	0.10	3.76E−17	0.54	0.22	1.58E−02
Other disorders of fluid, electrolyte and acid–base balance (ICD10)	− 0.85	0.11	4.11E−14	0.39	0.17	2.38E−02
Sheltered accommodation	− 0.85	0.12	3.37E−13	0.52	0.18	3.11E−03
Reduced smoking due to illness	− 0.82	0.12	7.92E−12	0.61	0.24	1.08E−02
Neck or shoulder pain for 3 + months	− 0.82	0.14	4.63E−09	0.70	0.16	1.16E−05
Entropion and trichiasis of eyelid	− 0.94	0.16	5.72E−09	0.43	0.18	1.88E−02
Self-reported type 2 diabetes	− 0.81	0.14	9.44E−09	0.45	0.15	2.05E−03
Polyarthrosis (ICD10)	− 0.69	0.13	3.07E−08	0.52	0.23	2.27E−02
Medication: enalapril	− 0.76	0.16	1.69E−06	0.26	0.09	5.34E−03
Medication: felodipine	− 0.77	0.17	8.22E−06	0.28	0.11	1.56E−02
Other arthrosis (ICD10)	− 0.74	0.17	1.90E−05	0.38	0.15	9.62E−03
Manifestations of mania or irritability: Being easily distracted	− 0.72	0.17	3.21E−05	0.38	0.09	3.92E−05
Medication: gabapentin	− 0.71	0.19	2.35E−04	0.47	0.13	2.73E−04
Other diseases of anus and rectum (ICD10)	− 0.69	0.19	2.86E−04	0.32	0.11	4.07E−03
Obesity	− 0.73	0.21	4.88E−04	0.86	0.21	4.40E−05
Disorders of synovium and tendon	− 0.82	0.23	4.92E−04	0.31	0.11	3.63E−03
Shift work	− 0.73	0.22	8.39E−04	0.79	0.10	1.04E−14
Medication: pantoprazole	− 0.70	0.21	9.56E−04	0.39	0.13	3.34E−03
Fibroblastic disorders	− 0.67	0.21	1.50E−03	0.19	0.07	6.13E−03
Unable to work due to sickness or disability	− 0.67	0.22	2.44E−03	0.48	0.16	2.53E−03
Syncope and collapse (ICD10)	− 0.74	0.24	2.52E−03	0.28	0.09	2.23E−03
Diseases of the skin and subcutaneous tissue	− 0.67	0.23	3.36E−03	0.53	0.11	1.59E−06

This table shows significant (FDR < 5%) traits with a robust and negative genetic causal proportion (GCP < − 0.60) with ADHD. Due to space constraints, results for all nominally significant genetic correlations for ADHD are shown in Supplementary File 1. *Trait* Trait causally associated with ADHD, *GCP* Genetic causal proportion, *GCP se* Genetic causal proportion standard deviation, Genetic causal proportion, *GCP pval* Genetic causal proportion p-value before FDR correction, *r*_*G*_ Genetic correlation, *r*_*G*_* se* Genetic correlation standard deviation, *r*_*G*_* p val* Genetic correlation p-value before FDR correction. P-values after FDR correction are available for all traits in Supplementary File 1.

Genetic variants associated with the frequent use of various medications as an adult were identified to contribute to a higher ADHD risk as a child. These medications included *Enalapril* (r_G_ = 0.79, GCP = − 0.73, GCP_pvalue_ = 1.69 × 10^−06^) and *Felodipine* (r_G_ = 0.28, GCP = − 0.77, GCP_pvalue_ = 8.22 × 10^−06^) which are typically prescribed for treating hypertension, *Gabapentin* (r_G_ = 0.47, GCP = − 0.71, GCP_pvalue_ = 2.35 × 10^−04^) prescribed for seizures and epilepsy*,* and *Pantoprazole* (r_G_ = 0.39, GCP = − 0.70, GCP_pvalue_ = 9.56 × 10^−04^) commonly prescribed for gastro-oesophageal reflux disease.

## Discussion

In the present study, we performed a large-scale genetic investigation of the potential causal architecture of ADHD risk. We used GWAS summary data to conduct bivariate LCV analyses between ADHD and 1,387 other phenotypes. As it has been previously stressed^10,11^, we note that both LCV and Mendelian randomisation methods test the effect of the genetic liability for a given phenotype on the outcome. For instance, potential genetic evidence of causal associations in the present study indicates the inferred causal effect between genetic variants that influence complex traits as an adult and ADHD risk as a child. Our results reveal that the genetic liability to multiple complex traits, notably cardiometabolic and pain-related phenotypes as an adult, increases the risk for ADHD as a child.

It is important to triangulate findings from studies with different study designs, of which at least one should be an interventional study such as a randomised controlled trial. However, interventional studies are known to be expensive, time-consuming, and sometimes unfeasible due to ethical concerns (i.e., evaluating an exposure known to harm participants). Thus, using methods in statistical genetics to identify the potential causal effect of genetic liability to a disease on an outcome could be the best option available, particularly for young- and late-onset phenotypes. Our findings contribute to elucidate the complex aetiology of ADHD and should be interpreted as a set of testable hypotheses for future studies.

A higher risk for *self-reported COPD* was causally influenced by genetic variants associated with ADHD*.* Previous studies have reported an association between ADHD and COPD^12,13^, and some suggest that genetic influences could partially explain this relationship^14^. However, it is well established that smoking is a risk factor for COPD^15,16^, and youth diagnosed with ADHD are two to three times more likely to engage in smoking behaviour^17^. We hypothesise that the association between ADHD and COPD could be influenced by potential vertical pleiotropic effects and may also be mediated by susceptibility to lifestyle factors.

Previous studies have assessed the relationship between iron deficiency and ADHD^18–20^, suggesting that iron deficiency may contribute to the physiopathology of ADHD by influencing dopaminergic dysfunction^21^. Our results show the potential causal effect of the genetic liability for *iron deficiency anaemia (ICD10)* on a higher risk for ADHD. Thus, we provide convergent evidence with previous studies suggesting that children with ADHD (or with a high ADHD predisposition) and low iron levels could benefit from iron supplementation. Nonetheless, an interventional, and preferably, a randomised clinical trial is required to confirm this hypothesis. For example, case–control studies could screen children with ADHD and iron deficiency to investigate if and to what extent iron supplementation in those with iron deficiency could prevent or ameliorate ADHD symptoms in the longer term.

It has been observed that obesity can be comorbid with ADHD^22^, and previous studies suggest that this relationship is not influenced by confounding factors^23^. Some genetic studies show a predominant one-way causal association in which obesity is potentially causal for ADHD^24,25^, while others suggest that there may be plausible bidirectional effects between ADHD and obesity^26^. In addition, it has been reported that children with diabetes are more likely to develop ADHD, even after accounting for possible confounding factors such as obesity^27^. However, the biological mechanisms behind this association remain unclear^27,28^. Similarly, some children with high blood pressure might have a comorbid diagnosis of ADHD^29,30^. In the present study, genetic liability for *obesity* appeared to lead to a higher risk for ADHD, as did genetic variants associated with other cardiometabolic traits such as *peripheral artery disease, self-reported type 2 diabetes,* and the use of *Enalapril* and *Felodipine*, which could be used as a proxy for hypertension*.* Consistently, we identified that genetic variants associated with low HDL cholesterol levels in adulthood increase the risk for ADHD in childhood; this is most likely explained by the effect of obesity-related variants, which are known to influence a decrease in HDL cholesterol^25,31,32^. Although we cannot rule out a potential influence of obesity in the potential causal association between cardiometabolic phenotypes and ADHD, our results suggest that genetic variants influencing the likelihood of *peripheral artery disease, hypertension,* and *type 2 diabetes* as an adult could also be partly responsible for a higher ADHD risk as a child.

Widespread musculoskeletal pain and motor inhibition problems have been observed among adults with ADHD^33,34^, and previous studies report that ADHD symptoms correlate with severe pain that hinders an individual’s capacity to work^34^. In our study, genetic liability for musculoskeletal pain-related phenotypes as an adult contributed to a higher ADHD risk as a child, whereas genetic susceptibility to ADHD increases the risk for *carpal tunnel syndrome*, which is commonly due to high levels of gaming/computer use in poor ergonomic setups^35,36^. Our results add up to the evidence suggesting that genetic risk variants for pain and poor musculoskeletal system health increase the risk for ADHD.

It has been reported that children with epilepsy are more likely to experience concentration problems^37^. ADHD’s prevalence among children with epilepsy ranges from 20 to 50%^38^. In this study, we showed genetic variants potentially influencing the use of *Gabapentin,* which could be used as a proxy for epilepsy^39^, and *syncope and collapse (ICD10),* posing a potential causal genetic effect increasing the risk for ADHD. Thus, our results support putative vertical pleiotropic effects between genetic susceptibility to epilepsy and ADHD.

The relationship between substance use and ADHD has become a focal point of interest in identifying risk factors for ADHD. Previous studies have reported that alcohol abuse is associated with the worsening of ADHD symptoms^40^. Also, it has been suggested that individuals with ADHD may be more sensitive to experience impairment effects of alcohol, particularly for inhibitory control^41^. Similarly, previous studies have identified associations between poor social skills and ADHD^42,43^, suggesting that interventions to refine social skills among individuals with ADHD could be of benefit^44^. In the present study, genetic evidence suggested that alcohol misuse as an adult may increase the risk for ADHD as a child. Consistently, genetic variants influencing the likelihood of *never being injured or having injured someone else through drinking alcoho*l were negatively correlated with ADHD risk, as were those for doing *unpaid or voluntary work*. Therefore, our results suggest that children with ADHD may be more likely to engage in substance use (particularly alcohol) as adults.

ADHD, bipolar disorder, major depressive disorder and schizophrenia are known to share common genetic risk^45^. Here, we observed that genetic correlations between ADHD and bipolar disorder, schizophrenia, anxiety and depression can be explained by horizontal pleiotropic effects rather than a potential causal pathway. Although psychiatric phenotypes may share considerable common variant genetic risk, we speculate that the spectral nature of psychiatric phenotypes may influence phenotypic overlap between them^45^, which in turn would contribute to the shared genetic risk among them.

The LCV method is known to have advantages over other traditional methods used in genetic epidemiology to investigate potential causal associations. These include that (a) it is less susceptible to bias due to horizontal pleiotropy^25,46,47^, (b) it can cope with sample overlap^25,46,47^ and (c) it increases statistical power by using information across the whole genome^25,46,47^, which in turn enables researchers to test potential causality with phenotypes that would be considered “underpowered” with other statistical methods.

Limitations of the present study include: (i) the generalisability of our results across ethnicities may be limited, given reports of ethnic differences in ADHD manifestations^48,49^. (ii) Although more than 1,300 phenotypes were included in our analysis, potential causal genetic effects with other traits may exist. (iii) Many GWAS used in this study may proxy other complex traits complicating the interpretability of the results. For instance, medication use GWAS were interpreted as a proxy for the phenotype they are most commonly prescribed for. (iv) The LCV method identifies the predominant causal pathway between a pair of correlated phenotypes and it is unable to test for bidirectional causality^8^. (v) Despite increased statistical power due to the use of aggregated genetic information throughout the genome, the LCV method still depends on the statistical power of the original GWAS^8^, and the capacity to identify potential causal genetic effects could be limited for some phenotypes, particularly for those with small sample sizes. Related to this is the inconsistency between obesity-related phenotypes. For example, *Diagnoses—main ICD10: E66 Obesity* refers to unspecified obesity diagnosed in the International Classification of Diseases. In contrast, *Obesity* refers to the combination of several ICD10 codes for obesity, including drug-induced obesity, morbid obesity with alveolar hypoventilation and obesity due to excess calories, among others. In this study, genetic susceptibility to both obesity phenotypes contributed to a higher risk of ADHD (Supplementary File 1); however, only *Obesity* survived multiple testing correction. This inconsistency is explained by differences in sample size and statistical power between the different GWAS. (vi) If several latent factors mediate a causal association between two traits, the LCV method may produce spurious findings, and GCP estimates could be biased towards the null due to a reduction of statistical power^8^.

It is important to note the temporality difference of phenotype measurements between ADHD and the phenotypes included in this large-scale study. ADHD symptoms are known to begin during childhood and are estimated to continue in adulthood for around 50% of those affected. We note that our results reflect the potential causal effect of genetic liability for a number of adult phenotypes on higher ADHD risk in childhood. Therefore, our results should be interpreted as a set of testable hypotheses that need to be validated in future investigations in both children and adults. For instance, longitudinal studies could monitor participants from childhood to adulthood to assess whether adults who had an ADHD diagnosis during childhood are more likely to present a clinical manifestation for a phenotype whose genetic liability was identified to increase the risk for ADHD in the present study. Also, given that the genetic liability for participating in socially supportive and interactive activities (i.e., volunteering) in adulthood is inversely correlated with high ADHD risk, future studies could investigate if and to what extent could these activities help refine social skills or manage ADHD symptoms in adults who had an ADHD diagnosis in childhood.

Here, we assessed the evidence for potential causal genetic effects between ADHD and more than 1,300 phenotypes. Our findings uncovered the potential role of iron metabolism in ADHD’s aetiology, supporting the hypothesis that iron supplementation could benefit children at high ADHD risk. Similarly, we identified the putative causal effect of the genetic susceptibility to substance use behaviours as an adult on a higher risk for ADHD as a child. Further, we show the probable influence of cardiometabolic phenotypes and poor musculoskeletal health as an adult on an increased risk for ADHD. Although the mechanisms are unclear, we highlight a possible role of genetic susceptibility to ADHD as a contributor to COPD. Altogether, our results contribute to our understanding of ADHD’s aetiology while supporting evidence from several previous observational studies and providing a set of novel testable hypotheses that need further validation.

## Methods

### ADHD dataset

We leveraged a large and publicly available GWAS summary statistics dataset for ADHD from the PGC. A detailed description of these summary statistics is available in their corresponding publication^6^. Briefly, an inverse variance weighted GWAS meta-analysis of childhood ADHD classified under DSM-IV was performed on samples from the PGC and the Lundbeck Foundation Initiative for Integrative Psychiatric Research (iPSYCH), including a total of 55,374 participants (20,183 cases and 35,191 controls). The Ricopilli pipeline^50^, developed by the PGC, was used to conduct stringent quality control and imputation procedures^6^. For each cohort, principal components were included as covariates in the model to control for population stratification^6^.

### CTG-VL datasets

A compilation of GWAS summary statistics for 1,387 polygenic traits and diseases are publicly available in The Complex Traits Genomics Virtual Lab (CTG-VL; https://genoma.io/)^51^. Details for these summary statistics are described directly in the CTG-VL. Briefly, summary statistics available in the CTG-VL include objective laboratory measurements and self-reported phenotypes from Neale’s Lab second wave of GWAS results from the UK Biobank cohort (www.nealelab.is/uk-biobank/)^52^ and several other GWAS consortia. Most GWAS are derived from European ancestry samples, mitigating potential biases due to population differences in linkage-disequilibrium and allele frequencies. The CTG-VL’s inclusion criteria require a nominally significant heritability derived from LD score regression^51^.

### Genetic causal proportion

We employed the phenome-wide analysis pipeline in the CTG-VL as described in previous studies^25,46,47^ to estimate genetic correlations using LD-score regression and perform bivariate latent causal variable (LCV) analysis between ADHD and 1,387 phenotypes to assess whether a genetic correlation (rG) could be explained by vertical pleiotropic effects (i.e., the effect of a genetic variant on a trait is mediated by its effect on another trait). Briefly, we loaded the ADHD GWAS summary statistics onto the CTG-VL and estimated genetic correlations and potential causal associations with 1387 other traits using the MASSIVE phenome-wide analysis pipeline. Then, we generated causal architecture plots to visualise the results. A detailed and illustrated description of this approach is available in previous studies^46,47^.

The CTG-VL uses the same scripts that the original authors of the LCV^8^ method made available in a GitHub repository (https://github.com/lukejoconnor/LCV) to implement the phenome-wide analysis pipeline in R 4.0.0^46^. The LD score script munge_sumstats.py was used to format data and ensure consistency of alleles and variants across GWAS summary statistics. HapMap 3 SNPs were extracted with the list of SNPs (w_hm3.snplist) (https://github.com/bulik/ldsc/wiki).

The LCV method mediates the relationship between two genetically correlated phenotypes with a latent variable *L*, representing the causal component between both traits, and estimates the genetic causal proportion parameter (GCP)^8^. A GCP value equal to zero indicates that horizontal pleiotropic effects mediate the genetic correlation and thus, provides no evidence for genetic causality between the phenotypes^8^. In contrast, an absolute GCP value equal to one represents full genetic causality^8^. An absolute GCP value below 0.60 represents a weak causal association and indicates limited partial genetic causality^8^. In the present study, LD score regression^53^ was used to estimate genetic correlations between ADHD and 1,387 other phenotypes. Bivariate LCV analyses were performed between ADHD and 578 traits with a significant genetic correlation. We applied Benjamini-Hochberg’s False Discovery Rate (FDR < 5%) adjustment to define statistical significance (adjusted p < 0.05) at both the genetic correlation and LCV steps.

### Ethics declarations

This study was approved by the Human Research Ethics Committee of the QIMR Berghofer Medical Research Institute. We confirm that all methods were performed in accordance with relevant guidelines and regulations.

### Consent to participate

Informed consent was obtained from all individual participants included in the study.

### Consent to publish

All participants provided informed consent for the publication of study results.

## Supplementary Information

Supplementary Legends.

Supplementary Information.

## Data Availability

Summary-level data used in the present study is publicly available at the Complex Traits Genomics Virtual Lab (https://genoma.io/) platform. Summary-level data for ADHD is publicly available at the Psychiatric Genomics Consortium website (https://www.med.unc.edu/pgc/shared-methods/open-source-philosophy/).
