# Supplementary material for: Large-scale genetic investigation reveals genetic liability to multiple complex traits influencing a higher risk of ADHD
Source: Sci Rep. 2021 Nov 19;11:22628. doi: 10.1038/s41598-021-01517-7 (PMC8604995; doi:10.1038/s41598-021-01517-7)
Supplement: Supplementary file 1 — Supplementary Legends. [file 41598_2021_1517_MOESM1_ESM.docx]

**SUPPLEMENTARY FILES**

**Supplementary File 1.** LCV output for ADHD.
